# Active Epilepsy and Seizure Control in Adults — United States, 2013 and 2015

**DOI:** 10.15585/mmwr.mm6715a1

**Published:** 2018-04-20

**Authors:** Niu Tian, Michael Boring, Rosemarie Kobau, Matthew M. Zack, Janet B. Croft

**Affiliations:** ^1^Division of Population Health, National Center for Chronic Disease Prevention and Health Promotion, CDC; ^2^Cutting Edge Technologies and Solutions (Cetechs), Mesa, Arizona.

Approximately 3 million American adults reported active epilepsy* in 2015 (*1*). Active epilepsy, especially when seizures are uncontrolled, poses substantial burdens because of somatic, neurologic, and mental health comorbidity; cognitive and physical dysfunction; side effects of antiseizure medications; higher injury and mortality rates; poorer quality of life; and increased financial cost (*2*). Thus, prompt diagnosis and seizure control (i.e., seizure-free in the 12 months preceding the survey) confers numerous clinical and social advantages to persons with active epilepsy. To obtain recent and reliable estimates of active epilepsy and seizure control status in the U.S. population, CDC analyzed aggregated data from the 2013 and the 2015 National Health Interview Surveys (NHISs). Overall, an annual estimated 2.6 million (1.1%) U.S. adults self-reported having active epilepsy, 67% of whom had seen a neurologist or an epilepsy specialist in the past year, and 90% of whom reported taking epilepsy medication. Among those taking epilepsy medication, only 44% reported having their seizures controlled. A higher prevalence of active epilepsy and poorer seizure control were associated with low family income, unemployment, and being divorced, separated, or widowed. Use of epilepsy medication was higher among adults who saw an epilepsy specialist in the past year than among those who did not. Health care and public health should ensure that adults with uncontrolled seizures have appropriate care and self-management support in order to promote seizure control, improve health and social outcomes, and reduce health care costs.

NHIS is an annual, nationally representative household survey of the U.S. civilian, noninstitutionalized population.^†^ Epilepsy data were collected in the NHIS Sample Adult component, which includes one randomly selected adult aged ≥18 years from each randomly selected household. In 2013, 34,557 adults (61.2% final response rate) responded to the survey, and in 2015, 33,672 adults (55.2% final response rate) responded.^§^ Data for 2013 and 2015 were aggregated to provide more reliable estimates (58.2% combined response rate). After excluding respondents with missing information on epilepsy history, 68,174 (99.9%) respondents were included in the analysis.

Adult respondents answered three questions about epilepsy to identify persons with active epilepsy and one question regarding specialty care.^¶^ These case-ascertainment questions have been validated for use in community surveillance (*3*). Prevalence of active epilepsy and percentages of respondents with epilepsy who had seen a neurologist or epilepsy specialist in the past year, who were taking epilepsy medication, and whose seizures were controlled (i.e., had no seizures during the past year) among those taking epilepsy medication were estimated for each survey year, both survey years, overall, and by selected sociodemographic characteristics. The percentages of adults taking epilepsy medication and the distribution of seizure frequencies among those with active epilepsy by epilepsy specialty care were also estimated. Prevalences and percentages were age-standardized to the projected 2000 U.S. adult population by four age groups: 18–34, 35–54, 55–64, and ≥65 years. Unless otherwise noted, the relative standard error of all estimates was <30.0%. Statistical software that accounted for the respondent sampling weights and the NHIS complex sample design was used for analysis. All reported differences between subgroups were statistically significant (p<0.05 by two-tailed t-tests).

During 2013 and 2015, the annual prevalence of active epilepsy was 1.1% (approximately 2.6 million adults) and was significantly higher in 2015 (1.2%) than in 2013 (0.9%). The age-adjusted prevalence of active epilepsy was significantly higher among respondents who were non-Hispanic white (white) and non-Hispanic black (black); never married, divorced, separated, or widowed; had less than high school diploma; were unemployed, or living in lower-income families (e.g., families earning <200% of federal poverty level [FPL]) than among other groups (Table).

**TABLE:** Number and age-adjusted* prevalence of active epilepsy, and percentages of adults who accessed specialty care, took epilepsy medications for seizure control, and were seizure-free with epilepsy medication in the past year among doctor-diagnosed active epilepsy,^†^ by selected characteristics — National Health Interview Survey, United States, 2013 and 2015

Characteristic	Adults with active epilepsy			Seen a neurologist or epilepsy specialist			Taking epilepsy medication to control seizure			Seizure-free with epilepsy medication		
	No.	No. (weighted)^§^	Age-adjusted % (95% CI)	No.	No. (weighted)^§^	Age-adjusted % (95% CI)	No.	No. (weighted)^§^	Age-adjusted % (95% CI)	No.	No. (weighted)^§^	Age-adjusted % (95% CI)
**Survey year**												
2013	367	2,254,000	0.9 (0.8–1.1)	217	1,428,000	65.7 (59.1–71.8)	305	1,948,000	86.3 (81.1–90.3)	136	871,000	45.3 (37.3–53.6)
2015	401	2,978,000	1.2 (1.1–1.4)	255	2,032,000	68.3 (62.1–74.0)	352	2,749,000	93.0 (89.8–95.3)	152	1,184,000	42.4 (35.0–50.2)
Total (crude)	768	2,616,000	1.1 (1.0–1.2)	472	1,730,000	66.2 (61.6–70.5)	657	2,348,000	90.2 (87.4–92.4)	288	1,028,000	44.1 (38.7–49.7)
Total (age-adjusted)	768	2,616,000	1.1 (1.0–1.2)	472	1,730,000	67.0 (62.6–71.2)	657	2,348,000	90.2 (87.4–92.4)	288	1,028,000	43.7 (38.1–49.5)
**Sex**												
Men	354	1,327,000	1.1 (1.0–1.3)	224	919,000	69.7 (62.9–75.7)	313	1,221,000	92.0 (87.5–95.0)	146	538,000	43.7 (36.1–51.5)
Women	414	1,289,000	1.0 (0.9–1.2)	248	811,000	64.5 (58.4–70.1)	344	1,128,000	88.3 (84.2–91.4)	142	490,000	43.5 (35.7–51.6)
**Age group (yrs)**												
18–34	165	803,000	1.1 (0.9–1.4)	116	615,000	76.7 (67.8–83.7)	136	721,000	91.0 (85.8–94.4)	54	303,000	42.3 (30.9–54.5)
35–54	280	867,000	1.0 (0.9–1.2)	175	585,000	67.5 (59.9–74.2)	235	768,000	88.6 (83.1–92.4)	80	280,000	36.9 (28.5–46.1)
55–64	165	540,000	1.4 (1.1–1.6)	95	305,000	56.5 (46.5–66.1)	144	483,000	89.4 (82.9–93.6)	63	209,000	43.7 (33.5–54.5)
>65	158	404,000	0.9 (0.7–1.1)	86	225,000	55.6 (45.0–65.7)	142	377,000	93.1 (88.0–96.1)	91	236,000	62.7 (50.7–73.3)
**Race/Ethnicity**												
White, non-Hispanic	507	1,857,000	1.2 (1.0–1.3)	306	1,246,000	67.9 (62.6–72.8)	440	1,692,000	91.4 (88.0–93.9)	216	811,000	47.5 (40.4–54.6)
Black, non-Hispanic	136	401,000	1.4 (1.1–1.7)	84	233,000	62.8 (50.6–73.7)	114	348,000	88.8 (81.0–93.6)	35	104,000	32.3 (21.5–45.3)
Other	125	357,000	0.7 (0.6–0.9)	82	251,000	70.5 (59.4–79.6)	103	309,000	86.5 (78.8–91.8)	37	113,000	37.3 (26.7–49.3)
**Marital status**												
Never married	246	934,000	2.0 (1.6–2.3)	165	691,000	71.0 (62.5–78.2)	219	862,000	92.7 (87.6–95.8)	87	351,000	44.9 (35.3–55.0)
Married/ Cohabitating	255	1,036,000	0.7 (0.6–0.8)	156	667,000	64.5 (56.4–71.9)	218	932,000	88.3 (82.5–92.3)	106	441,000	50.0 (41.5–58.5)
Divorced/ Separated/ Widowed	266	641,000	1.7 (1.2–2.3)	150	367,000	63.9 (53.4–73.2)	219	550,000	85.8 (76.6–91.8)	95	236,000	31.5 (25.3–38.4)
**Education level**												
Less than HS	194	564,000	1.8 (1.5–2.2)	104	310,000	58.7 (48.9–67.9)	164	498,000	89.4 (82.9–93.6)	63	192,000	39.0 (28.9–50.2)
HS diploma or GED	216	803,000	1.3 (1.1–1.6)	131	496,000	62.4 (53.5–70.5)	190	733,000	92.5 (88.0–95.4)	76	283,000	38.9 (30.2–48.4)
Some college	348	1,203,000	0.8 (0.7–0.9)	231	896,000	74.2 (67.8–79.8)	294	1,074,000	89.0 (84.3–92.4)	144	533,000	49.4 (41.3–57.6)
**Current employment**												
Yes	215	783,000	0.5 (0.4–0.6)	129	538,000	67.9 (59.4–75.5)	183	709,000	89.6 (83.8–93.5)	104	376,000	54.3 (44.1–64.1)
No	553	1,833,000	2.5 (2.2–2.8)	343	1,192,000	67.9 (62.6–72.7)	474	1,639,000	90.1 (86.7–92.8)	184	652,000	37.7 (31.0–45.1)
Poverty status**												
<200% of FPL	481	1,383,000	1.9 (1.6–2.1)	284	864,000	64.6 (58.4–70.4)	402	1,222,000	88.2 (84.0–91.4)	143	399,000	33.2 (26.6–40.4)
≥200% of FPL	287	1,233,000	0.8 (0.6–0.9)	188	866,000	70.6 (63.4–76.9)	255	1,126,000	92.1 (87.9–94.9)	145	628,000	55.3 (46.7–63.6)
**Region** ^††^												
Northeast	112	390,000	0.9 (0.7–1.2)	82	316,000	84.3 (74.9–90.7)	97	358,000	89.5 (79.7–94.9)	46	209,000	60.3 (47.1–72.1)
Midwest	157	549,000	1.0 (0.8–1.2)	94	349,000	63.9 (54.8–72.2)	136	491,000	90.1 (83.3–94.4)	67	233,000	47.9 (36.5–59.5)
South	291	1,096,000	1.2 (1.1–1.4)	177	706,000	66.7 (59.9–72.9)	251	977,000	90.3 (85.2–93.7)	98	374,000	37.5 (29.3–46.3)
West	208	580,000	1.0 (0.8–1.3)	119	359,000	61.5 (51.8–70.4)	173	522,000	89.3 (84.0–93.0)	77	212,000	41.4 (31.0–52.5)

**Abbreviations:** CI = confidence interval; GED = General Educational Development; HS = high school; FPL = federal poverty level.

* Age-adjusted to the 2000 U.S. projected population, aged ≥18 years, using four age groups: 18–34, 35–54, 55–64, and ≥65 years. All prevalence estimates are age-adjusted except those for age groups, and overall (crude).

^†^ Doctor-diagnosed active epilepsy was defined as having a diagnosis of epilepsy and either taking medication or having had one or more seizures in the past year, or both.

^§^ Annualized and weighted number rounded to 1,000s.

^¶^ Other race/ethnicity includes non-Hispanic American Indian and Alaska Native only; non-Hispanic Asian only; non-Hispanic Native Hawaiian and Pacific Islander only; and non-Hispanic multiple race.

** Poverty status was defined as the ratio of family income to FPL.

^††^ Northeast region (Connecticut, Maine, Massachusetts, New Hampshire, New Jersey, New York, Pennsylvania, Rhode Island, Vermont); Midwest region (Illinois, Indiana, Iowa, Kansas, Michigan, Minnesota, Missouri, Nebraska, North Dakota, Ohio, South Dakota, Wisconsin); South region (Alabama, Arkansas, Delaware, District of Columbia, Florida, Georgia, Kentucky, Louisiana, Mississippi, Maryland, North Carolina, Oklahoma, South Carolina, Tennessee, Texas, Virginia, West Virginia); West region (Alaska, Arizona, California, Colorado, Hawaii, Idaho, Montana, Nevada, New Mexico, Oregon, Utah, Washington, Wyoming).

The percentage of respondents with active epilepsy who had seen a neurologist or an epilepsy specialist in the past year was 67%. This percentage was significantly higher among respondents aged 18–34 years, with at least some college education, or who lived in the Northeast than that among respondents aged ≥55 years, who had less than a high school education, or who lived in other regions.

Ninety percent of respondents with active epilepsy took epilepsy medication, and this percentage did not significantly differ by sociodemographic characteristics. Among respondents taking epilepsy medication, 44% reported that their seizures were controlled in the past year. The prevalence of seizure control was significantly higher among adults aged ≥65 years (62.7%) than among those aged 35–54 years (36.9%); among persons who were married/cohabiting (50.0%) than among those who were divorced, separated, or widowed (31.5%); among persons who were employed (54.3%) than among those who were unemployed (37.7%); and among those with higher family incomes (≥200% of FPL; 55.3%) than among those from lower income households (<200% of FPL; 33.2%). By region, the prevalence of seizure control among respondents with epilepsy taking epilepsy medication who lived in the Northeast (60.3%) was significantly higher than those who lived in the South (37.5%).

Among adults with active epilepsy, the age-adjusted prevalence of taking epilepsy medication was higher among those who saw an epilepsy specialist in the past year (95.4%) than among those who did not (78.1%); however, seizure frequency among those with active epilepsy did not differ significantly between those who did and did not see a specialist in the past year (Figure).

**FIGURE:**
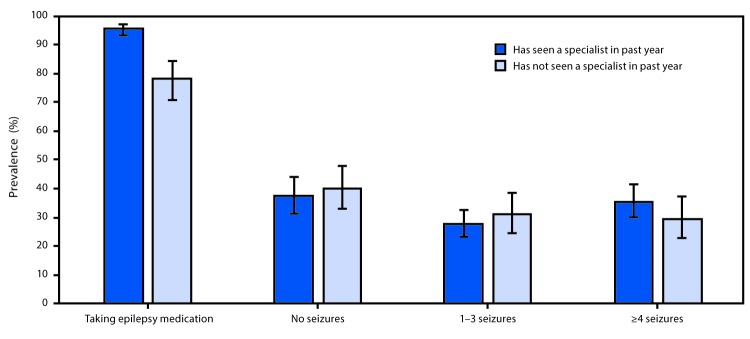
Age-standardized prevalence of epilepsy medication use and seizure frequency among adults with active epilepsy, by receipt of specialty care in the past year — National Health Interview Survey, United States, 2013 and 2015

## Discussion

The number of adults reporting that they have active epilepsy has significantly increased from 2010 (2.3 million) (*4*) to 2015 (3 million), with about 724,000 more cases identified from 2013 to 2015. In 2010, just over half (52.8%) of adults with active epilepsy saw a neurologist or epilepsy specialist (*4*). This study found that approximately two thirds (65.7% in 2013 and 68.3% in 2015) of adults with epilepsy saw a specialist. Most (90%) respondents with active epilepsy were taking epilepsy medication. Epilepsy medication use, but not reduced seizure frequency, was more common among those who had seen an epilepsy specialist; however, only 44% of respondents who took epilepsy medication had their seizures controlled in the past year. These results suggest that apart from the improvement associated with prompt diagnosis and treatment, other factors that might affect seizure control need to be addressed. The finding that blacks and respondents with less education and lower income had higher prevalences of active epilepsy is consistent with previous reports (*4*,*5*). This study also found that poor seizure control was associated with low income, unemployment, and being divorced, separated, or widowed. Socioeconomic disadvantage among adults with active epilepsy might preclude their accessing health care including specialty care (because of barriers such as cost and transportation) (*2*), thus affecting seizure control. More importantly, socioeconomic disadvantage (e.g., less education) and social isolation (e.g., lack of social support associated with being divorced, separated, or widowed) (*2*) might lead to nonadherence to epilepsy medication (*6*), an important clinical factor that significantly hinders seizure control (*6*,*7*).

Among adults taking epilepsy medication, those aged ≥65 years had better seizure control than among younger adults aged 35–54 years. This finding is also consistent with a previous report (*8*). The apparent better response to epilepsy medication in older adults might be attributable to differences in seizure etiology, drug pharmacokinetics, or better adherence to prescribed antiseizure medication regimens, possibly because of their experience with other chronic conditions or better access to care, including Medicare prescription drug coverage.

Only 44% of respondents with active epilepsy on epilepsy medication in this study were seizure-free in the past year. According to the Institute of Medicine, about 70% of all patients with epilepsy might become seizure-free under appropriate epilepsy treatment (*2*). To optimize seizure control, clinicians’ decisions to treat epilepsy should be based on individualized assessments of both disease-based (e.g., age of disease onset, seizure etiology, type, and comorbid conditions) and treatment-based factors (e.g., adherence to antiepileptic drugs), as well as patients’ personal characteristics, preferences, and their social context (*6*,*7*,*9*). Improving access to care, providing social support and epilepsy self-management education to improve medication adherence, and encouraging other self-management behaviors such as avoiding seizure triggers (e.g., sleep deprivation, stress, flashing lights, and alcohol or drug use) might also improve seizure control (*10*).

The findings in this report are subject to at least five limitations. First, estimates of epilepsy prevalence are based on self-reported data and are subject to error; however, because previous studies have validated the NHIS epilepsy questions, this bias is expected to be small (*3*). Second, active epilepsy might be overestimated because of the mistaken reporting of other nonepileptic seizures (*5*) or underestimated because of respondents’ reluctance to disclose epilepsy (*2*), as well as by the exclusion of institutionalized adults (e.g., adults in long-term care facilities and incarcerated persons) from NHIS. Third, these surveys did not objectively measure medication adherence or seizure frequency. Fourth, although respondent survey weights were adjusted to the U.S. population, the potential for nonresponse bias cannot be eliminated, given the low overall response rate (58.2%). Finally, the lack of differences in seizure frequency by seeing a specialist could be confounded by epilepsy severity and other untreated comorbidity such as mood disorder. However, no data regarding epilepsy severity is collected on NHIS

These findings highlight both the substantial burden of uncontrolled seizures in adults with epilepsy and the persistent sociodemographic and socioeconomic disparities in active epilepsy prevalence, access to neurologic specialty care, and seizure control. Health care and public health should ensure that adults with uncontrolled seizures have appropriate care and self-management support in order to promote seizure control, improve health and social outcomes, and reduce health care costs.

**Summary**

**What is already known about this topic?**

Approximately 3 million American adults have active epilepsy (doctor-diagnosed history of epilepsy, currently taking medication or having at least one seizure in the past year, or both). Uncontrolled seizures harm health, impair quality of life, and increase health care costs.

**What is added by this report?**

Although 90% of adults with active epilepsy were taking epilepsy medication, less than half (44%) of those taking medications were seizure-free in the past year. Seizures were more common among persons with lower household income, the unemployed, and the divorced, separated, or widowed.

**What are the implications for public health practice?**

Health care and public health should ensure that adults with uncontrolled seizures have appropriate care and self-management support in order to promote seizure control, improve health and social outcomes, and reduce health care costs.

## Footnotes

* Among those reporting that a doctor or health professional had told them they had a seizure disorder or epilepsy, those who reported taking medication, having had one or more seizures in the past year, or both were considered to have active epilepsy.

† https://www.cdc.gov/nchs/nhis/index.htm.

§ https://www.cdc.gov/nchs/nhis/data-questionnaires-documentation.htm.

¶ 1) “Have you ever been told by a doctor or other health professional that you have a seizure disorder or epilepsy?” 2) “Are you currently taking any medicine to control your seizure disorder or epilepsy?” 3) “Think back to last year about the same time. About how many seizures of any type have you had in the past year?” and 4) “In the past year have you seen a neurologist or epilepsy specialist for your epilepsy or seizure disorder?”

## References

[1] Zack MM, Kobau R. National and state estimates of the numbers of adults and children with active epilepsy—United States, 2015. MMWR Morb Mortal Wkly Rep 2017;66:821–5. 10.15585/mmwr.mm6631a1 28796763 PMC5687788

[2] Institute of Medicine. Epilepsy across the spectrum: promoting health and understanding. Washington, DC: The National Academy Press; 2012. https://www.nap.edu/read/13379/chapter/3#25

[3] Brooks DR, Avetisyan R, Jarrett KM, Validation of self-reported epilepsy for purposes of community surveillance. Epilepsy Behav 2012;23:57–63. 10.1016/j.yebeh.2011.11.002 22189155

[4] CDC. Epilepsy in adults and access to care—United States, 2010. MMWR Morb Mortal Wkly Rep 2012;61:909–13. 23151949

[5] Kroner BL, Fahimi M, Kenyon A, Thurman DJ, Gaillard WD. Racial and socioeconomic disparities in epilepsy in the District of Columbia. Epilepsy Res 2013;103:279–87. 10.1016/j.eplepsyres.2012.07.005 22858309 PMC4608437

[6] Szaflarski M. Social determinants of health in epilepsy. Epilepsy Behav 2014;41:283–9. 10.1016/j.yebeh.2014.06.013 24998313

[7] Garnett WR. Antiepileptic drug treatment: outcomes and adherence. Pharmacotherapy 2000;20:191S–9S. 10.1592/phco.20.12.191S.35250 10937819

[8] Stefan H, May TW, Pfäfflin M, Epilepsy in the elderly: comparing clinical characteristics with younger patients. Acta Neurol Scand 2014;129:283–93. 10.1111/ane.12218 24495079

[9] Manjunath R, Davis KL, Candrilli SD, Ettinger AB. Association of antiepileptic drug nonadherence with risk of seizures in adults with epilepsy. Epilepsy Behav 2009;14:372–8. 10.1016/j.yebeh.2008.12.006 19126436

[10] Schachter SC. Seizure disorders. Med Clin North Am 2009;93:343–51, viii. 10.1016/j.mcna.2008.10.001 19272512

